# Cholangiocarcinoma in a Child with Progressive Abdominal Distension and Secondary Hypercalcemia

**DOI:** 10.1155/2018/6828037

**Published:** 2018-04-23

**Authors:** Chalinee Monsereenusorn, Kantang Satayasoontorn, Piya Rujkijyanont, Chanchai Traivaree

**Affiliations:** ^1^Division of Hematology/Oncology, Department of Pediatrics, Phramongkutklao Hospital and College of Medicine, Bangkok, Thailand; ^2^Department of Pathology, Phramongkutklao Hospital and College of Medicine, Bangkok, Thailand

## Abstract

Cholangiocarcinoma is extremely rare in childhood and has been reported in association with other underlying diseases. The survival and prognosis are dismal especially in patients with unresectable or advanced stage cholangiocarcinoma. Overall survival in patients with metastatic cholangiocarcinoma could be increased by using combination chemotherapy with cisplatin and gemcitabine. A case of childhood cholangiocarcinoma was hereby reported.

## 1. Introduction

Cholangiocarcinoma is an adenocarcinoma from a bile duct that arises from the epithelial cells of the intrahepatic and extrahepatic bile ducts. These cancers are rare in the United States; on the contrary, in Asia, particularly Thailand, infection with liver flukes of the genera *Clonorchis* and *Opisthorchis* is often associated with cholangiocarcinoma of the intrahepatic bile ducts [1–3]. They are highly lethal because most of them are locally advanced or are metastatic at presentation.

Cholangiocarcinoma is most commonly diagnosed in the late eighth decade of life [4, 5]. It is exceedingly rare before adulthood. However, cholangiocarcinoma in childhood has been reported in association with HIV infection [6] and biliary atresia [7], following radiation therapy [8], as a sequela of a choledochal cyst [9, 10], primary sclerosing cholangitis [11, 12], and inflammatory bowel disease [12], and with primary immune deficiency [13].

A case of cholangiocarcinoma in an adolescent young adult has been reported.

## 2. Case Presentation

An 11-year-old Thai boy presented with progressive abdominal distension for one month prior to admission. He had a significant weight loss up to 8 kg within one month, but had no fever or jaundice. There is a history of eating habits: eating uncooked fish, meat, and beef. He also had polyuria upon admission. On physical examination, it was found that he was cachexic. His weight was in the 3rd percentile. Generalized lymphadenopathy and marked hepatosplenomegaly were detected. Neurological examination was within the normal limit.

**Figure 1:**
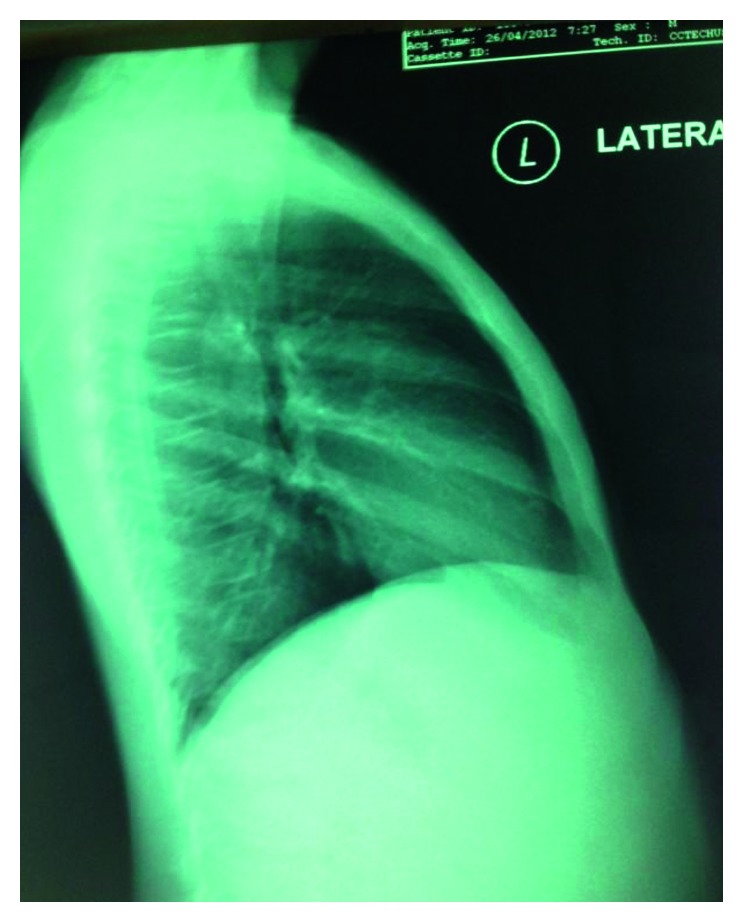
A chest X-ray showing no mediastinal mass and normal lung parenchyma. Generalized osteopenia was identified at the vertebral area.

His laboratory data revealed that the hemoglobin level was 13.3 g/dl, the hematocrit value was 41.5 volume %, the WBC count was 15,100 cells/mm^3^ (neutrophil 60%, lymphocyte 30%, monocyte 9%, and basophil 1%), and the platelets count was 304,000 cells/mm^3^. Peripheral blood smears were normochromic and normocytic red blood cells, without blasts cells. Bone marrow examination and biopsy were normal. The chromosome study reviewed 46 XY, normal male karyotype. The liver function test revealed that the level of albumin was 4.9 g/dl, total bilirubin 0.3 mg/dl, direct bilirubin 0.2 mg/dl, SGOT 73 U/L, SGPT 46 U/L, and alkaline phosphatase 339 U/L. Uric acid level was 6.1 mg/dl. The amount of lactate dehydrogenase (LDH) was 149 U/L. Serum electrolytes showed that the level of sodium was 134 mmol/l, potassium 3.82 mmol/l, chloride 98.5 mmol/l, and bicarbonate 26.3 mmol/l. Hypercalcemia and hypophosphatemia were detected; the serum calcium level had increased to 13.6 mg/dl and the phosphate level to 2.3 mg/dl. The blood urea nitrogen level was 11.7 mg/dl and creatinine 0.5 mg/dl. Serum amylase was 37 U/L, and serum lipase was higher than normal of 96 U/L. The amount of gamma-glutamyl transpeptidase (GGT) was 630 (normal range 15–85) U/L. Stool examination revealed blastocystis hominis. Chest X-ray showed generalized osteopenia (Figure 1). The bone scan showed no evidence of active bone lesion.

**Figure 2:**
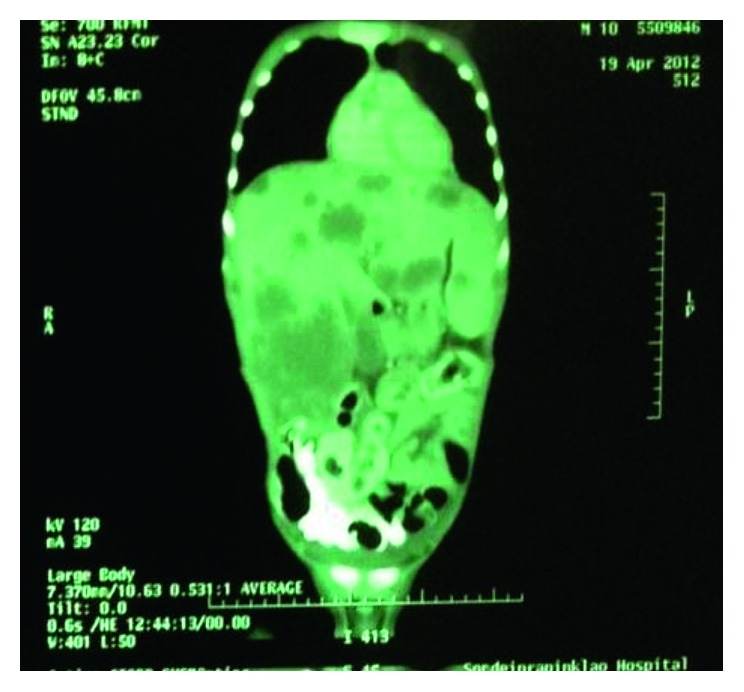
CT chest and whole abdomen (coronal view) showing marked hepatosplenomegaly, enlarged size of the pancreas, and numerous heterogeneous masses at both lobes of the liver and the pancreas.

**Figure 3:**
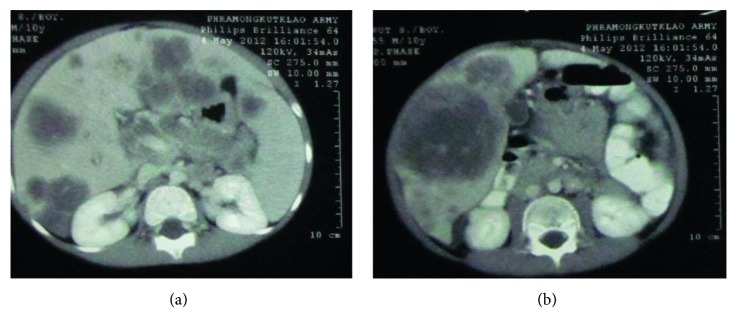
CT chest and whole abdomen (axial view). (a) Multiple masses at both lobes of the liver, the spleen, and the pancreatic body and tail. (b) The largest hepatic mass of 8 × 7.5 × 11.6 cm in diameter.

CT chest and whole abdomen (Figures 2 and 3) revealed hepatomegaly and numerous heterogeneous low-attenuation centers varying in size, with peripheral rim-enhancing masses scattered in both hepatic lobes, and the largest one was 8 × 7.5 × 11.6 cm in size. No dilatation of intrahepatic duct and CBD was seen. The spleen was enlarged, and the hypodensity mass size was 1.4 × 1.9 cm. The pancreas was enlarged with an ill-defined irregular hypodensity mass at the pancreatic body, and the tail size was 2.2 × 4.7 × 2.8 cm. Enlarged portal and para-aortic nodes were observed.

Investigations for hypercalcemia were performed. The intact parathyroid hormone (iPTH) level was very low 6.33 (normal range 15–65) pg/ml, while the 25-OH vitamin D level was 27 (normal range 30–100) ng/ml. It was shown that secondary hypercalcemia developed due to malignancy that is most likely from the parathyroid hormone- (PTH-) related protein (PTHrP) producing tumor.

The level of serum tumor markers such as alpha fetoprotein was 78.52 ng/ml, CA 19-9 was 360.8 U/ml, and CA 125 was 267.10 U/ml while the CEA and *β*-hCG were normal.

**Figure 4:**
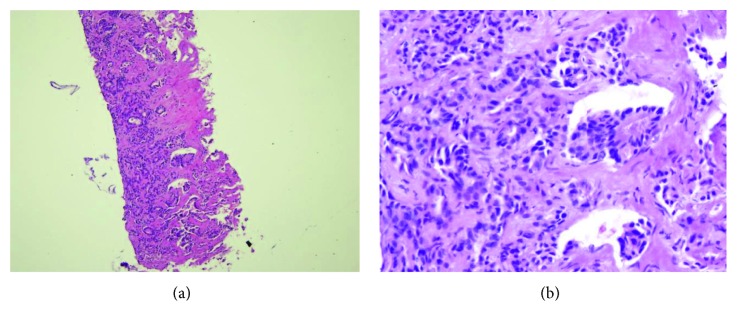
Liver biopsy revealing proliferation of atypical tubular elements associated with fibrous stroma characteristic of cholangiocarcinoma. H&E staining, original magnification: (a) ×10 and (b) ×40.

Liver biopsy was performed for diagnosis. Histological examinations of fine-needle biopsy showed poorly differentiated tubular adenocarcinoma (Figure 4).

**Figure 5:**
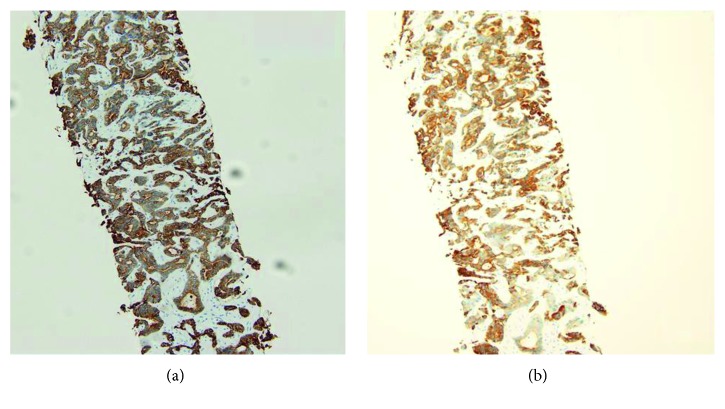
The bile canalicular immunohistochemical staining pattern in cholangiocarcinoma with diffuse positivity in cytoplasmic membrane staining of CK7 (a) and CK19 (b).

**Figure 6:**
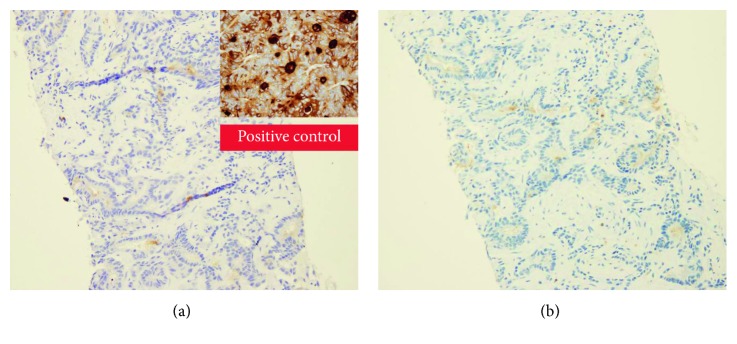
Immunohistochemical staining showing lack of the monoclonal CEA (a) and the polyclonal CEA (b).

In regard to immunohistochemistry, the tumor cells diffuse positive for cytokeratin 7 and 19 (Figure 5), negative for cytokeratin 20 and synaptophysin, and lacked mCEA and pCEA (Figure 6).

The patient was treated with vigorous hydration and diuretic; 20 mg of furosemide was given intravenously every 8 hours until the serum calcium level became lower than 12 mg/dl. He was placed on systemic chemotherapy; cisplatin (25 mg/m^2^) and gemcitabine (1,000 mg/m^2^) were administered intravenously on days 1 and 8 every 3 weeks for 8 cycles (24 weeks).

The patient was followed up with pediatric hemato-oncologists regularly. Physical examination and laboratory evaluation after the completion of the first cycle of chemotherapy revealed liver span, and the spleen decreased in size from 20 cm to 8 cm and 4 cm below left costal margin to just palpable. , respectively Also, the serum calcium level had decreased to 12.1 mg/dl, the phosphate level increased to 3.4 mg/dl, and the intact parathyroid hormone (iPTH) level had increased to 7.75 (normal range 15–65) pg/ml. However, he discontinued hydration therapy and diuretics.

Repeated CT chest and whole abdomen after the completion of the fourth cycle of chemotherapy for 12th and 24th weeks showed a decrease in size of the infiltrative mass in both hepatic lobes, the spleen, the pancreas, and intra-abdominal lymph nodes.

## 3. Discussion

Cholangiocarcinoma is extremely rare in childhood. The overall incidence rate is 0.0036 per 100,000 [14]. The youngest patient who had been reported was an 11-year-old male patient demonstrating cholangiocarcinoma associated with congenital biliary dilatation [10] presented with sporadic advanced metastatic stage at initial diagnosis [15]. Childhood cholangiocarcinoma patients are highly associated with congenital malformation of the bile duct system [10] or primary sclerosing cholangitis [11, 12], particularly in patients under age 20. There is no previous reports of patients who have no underlying disease of biliary tract, such as congenital biliary dilatation or primary sclerosing cholangitis [14]. Our patient in this report also does not have an underlying disease before diagnosis.

The patient in this report was at cholangiocarcinoma stage IV at initial diagnosis. The immune status of the patient should be evaluated with regard to primary immune deficiency, and immunologic disturbances such as hyper IgM syndrome has been reported in the literature review [13]. In patients with primary sclerosing cholangitis in whom cholangiocarcinoma develops, prognosis and therapy are dependent on whether the tumor is surgically resectable. In patients with unresectable cholangiocarcinoma, the prognosis is dismal [14], with a median survival of 9 to 12 months [16]. The survival rate after the onset of symptoms of unresected cholangiocarcinoma was 53% at 1 year, 19% at 2 years, and 9% at 3 years. Only four patients (4%) lived more than 5 years [17].

Hypercalcemia occurs in association with rhabdomyosarcoma, hepatoblastoma, some brain tumors, neuroblastoma, and hematologic cancers, including lymphoma and acute leukemia in children [18]. In general population, hypercalcemia associated with cancer mostly due to PTHrP in 80% population is found in squamous cell cancer [19–21].

Distinguishing hepatocellular carcinoma (HCC) from cholangiocarcinoma (CC) and metastatic adenocarcinoma (MA) involving the liver is always an issue, often requiring the use of immunohistochemistry to facilitate diagnosis. In cholangiocarcinoma, CK7 and CK19 are positive and are about 90–96% and 84%, respectively. The polyclonal CEA had high sensitivity of about 50–90% for hepatocellular carcinoma. The negative test can rule out hepatocellular carcinoma while the monoclonal CEA had low sensitivity of 60% in adenocarcinoma if the test was negative and therefore cannot rule out adenocarcinoma[22].

Chances of survival depend on disease resectability. Patients with metastatic disease have poor outcomes regardless of surgical resection. Three-year overall survival was 35–50% [14]. Overall survival in randomized 410 patients with metastatic cholangiocarcinoma comparing those receiving gemcitabine alone versus cisplatin plus gemcitabine was studied [23]. The patients treated with cisplatin plus gemcitabine lived an average of 3.6 months longer than those treated with gemcitabine alone.

In our case report, the patient was initially started with chemotherapy of cisplatin and gemcitabine regimen for 1-2 cycles. His clinical conditions in terms of urination and abdominal discomfort were improved. Repeated CT scan of the abdomen showed a dramatically shrunken mass, and the blood calcium was declined to the normal level. Because of the aggressiveness of the disease, after the completion of 6 cycles of chemotherapy, the tumor recurred, and the patient died due to progressive disease. His overall survival was 15 months.
